# Improvement in early urinary continence recovery after robotic-assisted radical prostatectomy based on postoperative pelvic anatomic features: a retrospective review

**DOI:** 10.1186/s12894-019-0519-8

**Published:** 2019-09-18

**Authors:** Akihiro Nakane, Hiroki Kubota, Yusuke Noda, Tomoki Takeda, Yasuhiko Hirose, Atsushi Okada, Kentaro Mizuno, Noriyasu Kawai, Keiichi Tozawa, Yutaro Hayashi, Takahiro Yasui

**Affiliations:** 1 Department of Urology, The Aichi Prefectural Federation of Agricultural Cooperatives for Health and Welfare Kainan Hospital, Yatomi, Japan; 2 Department of Nephro-urology, Nagoya City University Graduate School of Medical Sciences, 1 Kawasumi, Mizuho-cho, Mizuho-ku, Nagoya, 467-8601 Japan; 3 Department of Pediatric Urology, Nagoya City University Graduate School of Medical Sciences, Nagoya, Japan

**Keywords:** Robotic-assisted radical prostatectomy, Urinary incontinence, Pelvic anatomy, Magnetic resonance imaging

## Abstract

**Background:**

We investigated the impact of postoperative membranous urethral length and other anatomic characteristics of the pelvic floor shape as measured by magnetic resonance imaging on the improvement in continence following robotic-assisted radical prostatectomy.

**Methods:**

We retrospectively reviewed data from 73 patients who underwent postoperative prostate magnetic resonance imaging following robotic-assisted radical prostatectomy between 2013 and 2018. Patient demographics; pre-, peri-, and post-operative parameters; and pelvic anatomic features on magnetic resonance imaging were reviewed. Patients who used no urinary incontinence pads or pads for protection were considered to have achieved complete continence.

**Results:**

Urinary continence was restored in 27.4, 53.4, 68.5, and 84.9% of patients at 1, 3, 6, and 12 months after robotic-assisted radical prostatectomy, respectively. When patients were divided into early and late continence groups based on urinary continence at 3 months after robotic-assisted radical prostatectomy, no significantly different clinical characteristics or surgical outcomes were found. However, the mean membranous urethral length (18.5 mm for the early continence group vs. 16.9 mm for the late continence group), levator muscle width (7.1 vs. 6.5 mm, respectively), and bladder neck width on the trigone side (7.2 mm vs. 5.4 mm, respectively) were significantly different between groups (all *p* < 0.05). Multivariate logistic regression analysis showed that membranous urethral length (odds ratio, 1.227; 95% confidence interval, 1.011–1.489; *p* = 0.038) and bladder neck width (odds ratio, 1.585; 95% confidence interval, 1.050–2.393; *p* = 0.028) were associated with the period of early urinary continence.

**Conclusions:**

Postoperative membranous urethral length and bladder neck width were significantly associated with early urinary continence recovery after robotic-assisted radical prostatectomy. It is highly recommended that surgeons focus on preserving the membranous urethral length and increasing the bladder neck width on the trigone side during surgery to achieve optimal continence outcomes after robotic-assisted radical prostatectomy.

## Background

Robotic-assisted radical prostatectomy (RARP) has been widely used as the most advanced treatment for localized prostate cancer [1, 2]. Urinary incontinence after RARP arguably has the most significant impact on quality of life postoperatively [3], and 4–22% of patients reported not achieving urinary continence within 12 months after RARP [4–8]. Recent studies have reported that a patient’s preoperative status and certain anatomic characteristics are predictors of urinary continence postoperatively, therefore, it is indicated that surgical techniques and urinary incontinence after RARP are significantly related [6, 9–12]. The membranous urethral length (MUL) is an important factor that directly correlates with the sphincter’s functional mechanism. The urethral sphincter is composed of an external striated sphincter and an internal smooth muscle layer, both of which are important increasing urethral closure pressure [13]. Previous studies showed that MUL preservation is important because it relates to urinary continence after open or laparoscopic radical prostatectomy [9, 10]. Preoperative MUL has been shown to be an important predictor of urinary continence recovery [14]. Early recovery of urinary continence after RARP has been shown to depend on postoperative MUL [15]. However, MUL is almost completely determined by the anatomic characteristics of the patient’s original pelvic floor shape [14].

Therefore, we investigated the impact of postoperative MUL on urinary continence recovery after RARP and the association between MUL and other anatomic characteristics of the pelvic floor shape, as measured with magnetic resonance imaging (MRI), with incontinence improvement after RARP.

## Methods

### Patient characteristics

Between May 2013 and March 2018, 301 patients who underwent RARP for prostate cancer were retrospectively reviewed. RARP was performed using the da Vinci Si surgical robot (Intuitive Surgical, Inc., Sunnyvale, CA, USA). In all cases, the Rocco stitch was used for posterior reconstruction of the Denonvillier’s fascia [16, 17], and we did not routinely perform neurovascular bundle preservation, puboprostatic ligament preservation, and reconstruction of the distal apex. Postoperative pelvic MRI was performed on all patients who visited after RARP between May 2016 and November 2017, regardless of surgical procedure and pathological results, postoperative course. Postoperative pelvic MRI was routinely performed at 3 months postoperatively. Seventy-three patients who subsequently underwent postoperative pelvic MRI were identified. No patients had local recurrence or received salvage radiation therapy before undergoing postoperative pelvic MRI. None of these patients included neurovascular bundle preservation cases. All study protocols were approved by the Ethical Committee of the Aichi Prefectural Federation of Agricultural Cooperatives for Health and Welfare Kainan Hospital (approval no. 300214–01).

### Clinical and pathological parameters

Patient demographics (age and body mass index [BMI]), preoperative parameters (preoperative prostate-specific antigen [PSA] level and Biopsy Gleason’s score), perioperative parameters (operative time, console time, estimated blood loss, and prostate volume), and postoperative parameters (pathological T stage, positive surgical margin, catheter removal period, postoperative hospital stay, and postoperative continence status) were reviewed. A five-point scale was used to assess continence grade [10]. Patients with Level 1 who did not use urinary incontinence pads or pads for protection were considered to have achieved complete continence.

### MRI measurements

**Fig. 1:**
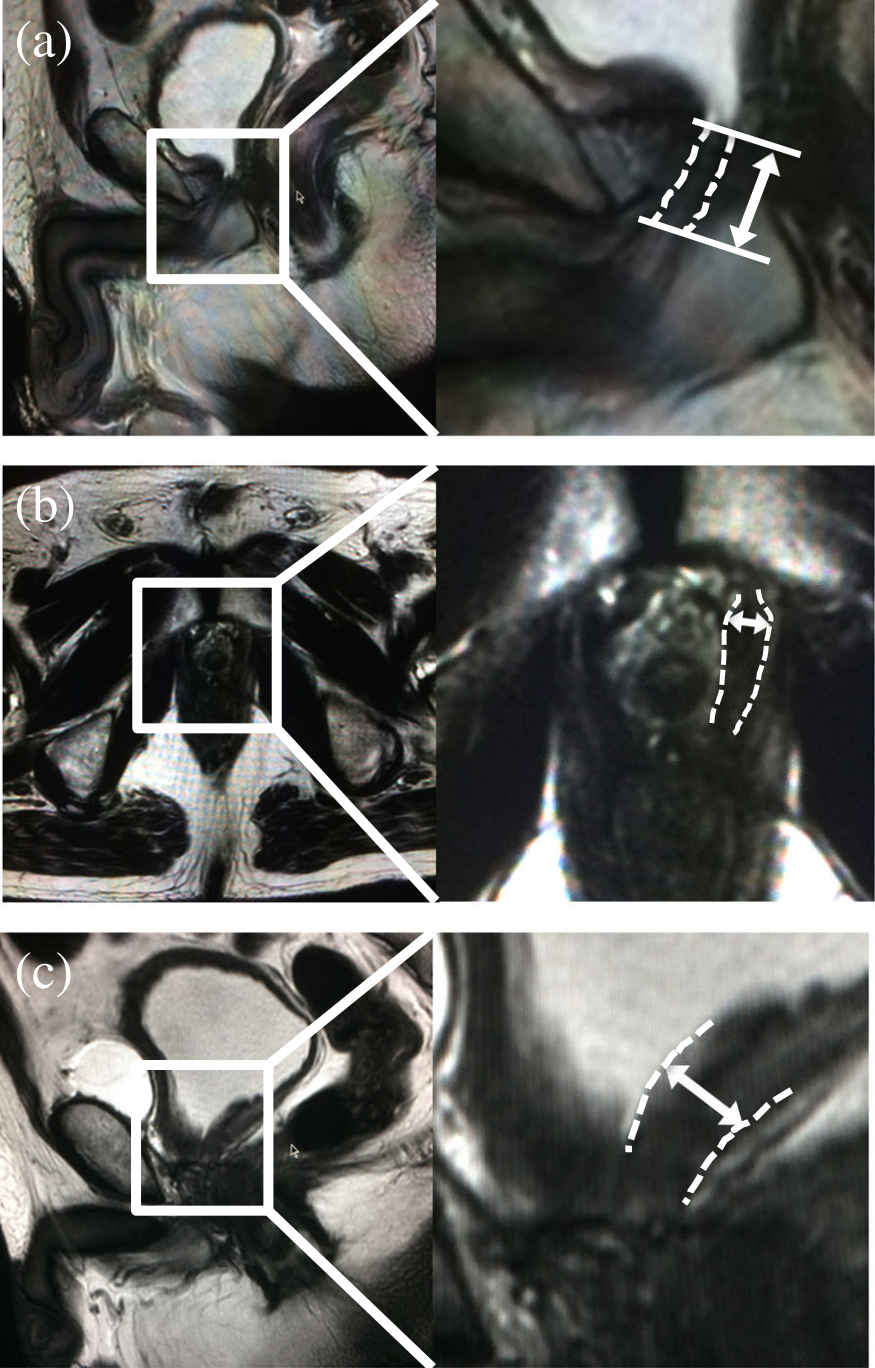
Postoperative pelvic anatomic features on T2-weighted magnetic resonance imaging. **a** Membranous urethral length measured in the sagittal plane. The dashed lines show the membranous urethra. **b** Levator muscle width measured in the axial plane. The dashed lines show the levator muscle. **c** Bladder neck width on the trigone side measured in the sagittal plane. The dashed lines show the posterior bladder neck wall

MRI was performed using a 1.5-T whole-body magnetic resonance scanner (Ingenia, Philips Healthcare, Best, the Netherlands). Images were obtained in 2 mm slices with T2-weighted sequences of the entire pelvis in the axial, sagittal, and coronal views. MUL was estimated in the midline sagittal plane (Fig. 1a). Levator muscle width (LMW) was estimated on axial images at the thickest portion of the urethral sphincter (Fig. 1b). Bladder neck width on the trigone side (BNW) was estimated on midline sagittal images (Fig. 1c).

### Statistical analysis

Continuous variables were expressed as means (standard deviation, range) and compared using the independent t-test. Multivariate logistic regression analyses were performed to determine predictive factors associated with early recovery of urinary continence after RARP. All statistical analyses were performed using SPSS Statistics Ver. 22 (IBM, Armonk, NY, USA). Two-sided *p* < 0.05 were considered statistically significant.

## Results

**Fig. 2:**
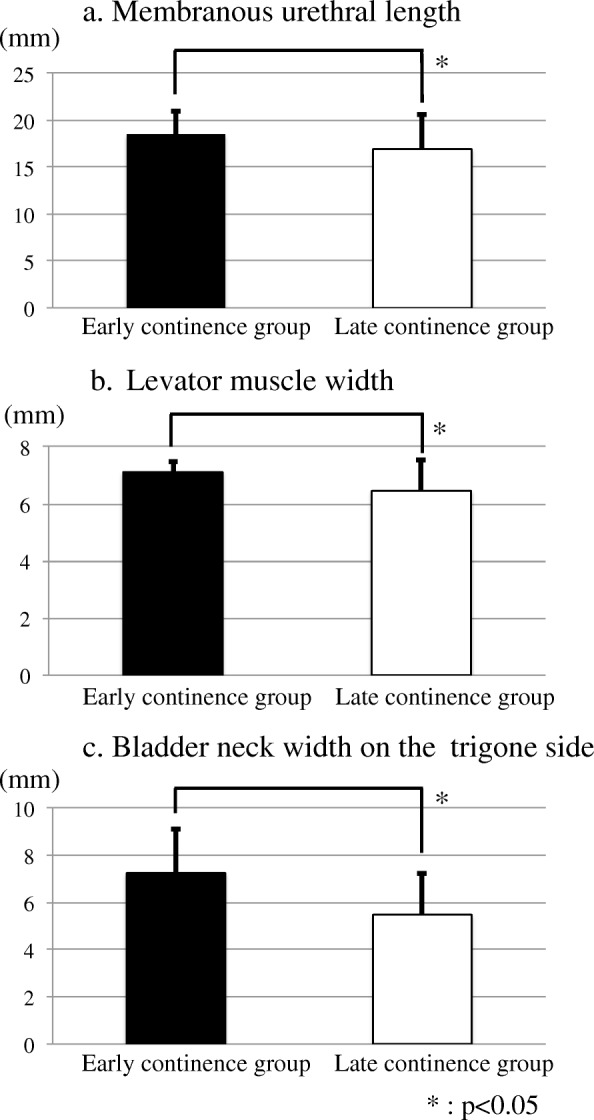
Association between postoperative pelvic anatomic features and urinary continence after robot-assisted radical prostatectomy. Pelvic anatomic features were evaluated with magnetic resonance imaging and compared based on postoperative urinary continence. **a** Membranous urethral length. **b** Levator muscle width. **c** Bladder neck width on the trigone side

Baseline clinical and pathological characteristics of the 73 patients who underwent RARP and postoperative MRI and 301 patients in the whole series are summarized in Table 1. At 1 month after RARP, urinary continence was restored in 27.4% of 73 patients who underwent RARP and postoperative MRI. Urinary continence improved over time, with 53.4, 68.5, and 84.9% of patients achieving urinary continence at 3, 6, and 12 months, respectively. When patients were divided into two groups (early versus late recovery of continence) based on urinary continence at 3 months after RARP, no significantly different clinical characteristics or surgical outcomes were observed (Table 2). We evaluated the association between postoperative pelvic anatomic features on MRI and urinary continence at 3 months (Fig. 2). The mean MUL (18.5 mm for the early continence group vs. 16.9 mm for the late continence group), levator muscle width (LMW) (7.1 mm for the early continence group vs. 6.5 mm for the late continence group), and BNW (7.2 mm for the early continence group vs. 5.4 mm for the late continence group) were significantly different between groups (all *p* < 0.05).

**Table 1:** Clinical characteristics and surgical outcomes

	Patients who underwent postoperative prostate MRI (*n* = 73)		Patients in the whole series (*n* = 301)	
Age (years; mean ± SD; range)	67.9 ± 5.4	(53–76)	68.7 ± 4.7	(53–76)
BMI (kg/m^2^; mean ± SD; range)	24.1 ± 2.8	(18.9–30.7)	23.9 ± 2.8	(16.8–33.4)
Preoperative PSA level (ng/mL; mean ± SD; range)	9.9 ± 6.9	(4.0–43.4)	9.5 ± 6.6	(4.0–57.1)
Clinical T stage (n, %)				
cT1	23 (30.3)		148 (49.2)	
pT2	49 (67.1)		136 (45.2)	
pT3	1 (1.4)		17 (5.6)	
Biopsy Gleason’s score (n, %)				
≦ 6	25 (34.2)		94 (31.2)	
= 7	30 (41.1)		137 (45.5)	
≧ 8	18 (24.7)		70 (23.3)	
Operative time (min; mean ± SD; range)	182.3 ± 48.8	(108–307)	187.3 ± 44.4	(103–313)
Console time (min; mean ± SD; range)	141.0 ± 41.7	(76–250)	145.6 ± 41.3	(74–289)
Estimated blood loss (mL; mean ± SD; range)	98.1 ± 76.6	(2–380)	93.3 ± 99.4	(1–650)
Prostate volume (mL; mean ± SD; range)	50.2 ± 24.2	(10–175)	44.1 ± 18.9	(10–175)
Pathological T stage (n, %)				
pT2	57 (78.1)		226 (75.1)	
pT3	16 (21.9)		75 (24.9)	
Positive surgical margins (n, %)				
All stages	11 (15.1)		62 (20.1)	
pT2	7 (12.3)		29 (12.8)	
pT3	4 (25.0)		33 (44.0)	
Catheter removal period (days; mean ± SD; range)	7.2 ± 0.8	(6–13)	7.5 ± 3	(4–45)
Postoperative hospital stay (days; mean ± SD; range)	10.7 ± 2.2	(8–20)	10.9 ± 4.9	(7–49)
% continence (%)				
≤ 1 month	27.4		31.7	
≤ 3 months	53.4		51.2	
≤ 6 months	68.5		70.3	
≤ 12 months	84.9		78.8	

*Abbreviations*: *BMI* Body mass index, *PSA* Prostate-specific antigen, *SD* Standard deviation

**Table 2:** Clinical characteristics and surgical outcomes for patients based on continence recovery at 3 months

	Early continece group	Late continece group
n	39	34
Age (years)	68.2 ± 6.2	67.6 ± 4.8
BMI (kg/m^2^)	23.8 ± 3.0	23.1 ± 2.4
Console time (min)	179.0 ± 41.7	157.3 ± 37.8
Prostate volume (mL)	39.8 ± 12.6	43.7 ± 12.7
Catheter removal period (days)	7.0 ± 0.0	7.1 ± 0.3
Postoperative hospital stay (days)	11.3 ± 2.9	10.3 ± 1.4

*Abbreviation*: *BMI* Body mass index

Because 64 patients reached complete urinary continence after RARP during the observation period, multivariate logistic regression analysis was used to identify prognostic MRI measurements predicting early complete urinary continence at 3 months after RARP. Multivariate logistic regression analysis evaluated clinical characteristics and perioperative parameters together with MRI parameters, and the results are summarized in Table 3. On the multivariate analysis, MUL (odds ratio [OR], 1.227; 95% confidence interval [CI], 1.011–1.489; *p* = 0.038) and BNW (OR. 1.585; 95% CI 1.050–2.393; *p* = 0.028) were associated with the period of early complete urinary continence.

**Table 3:** Multivariate logistic regression analysis results for clinical characteristics, Perioperative parameters, and MRI measurements predicting the early urinary complete continence at 3 months after RARP

Clinical characteristics	Univariate analysis			Multivariate analysis		
	OR	95% CI	*p*-value	OR	95% CI	*p*-value
Age	0.924	0.841, 1.014	0.096	1.007	0.885, 1.146	0.912
BMI	1.110	0.932, 1.323	0.242	1.018	0.803, 1.290	0.884
Clinical T stage (cT1, cT2, cT3)	1.525	0.547, 4.248	0.420	2.354	0.469, 11.806	0.298
Biopsy Gleason’s score (**≦**6, 7, 8**≦**)	0.813	0.456, 1.449	0.483	0.741	0.289, 1.904	0.534
Perioperative parameters						
Prostate volume	1.003	0.983, 1.023	0.783	0.996	0.965, 1.028	0.807
Catheter removal period	0.843	0.451, 1.576	0.593	1.001	0.510, 1.963	0.999
Postoperative hospital stay	0.955	0.767, 1.191	0.685	0.870	0.654, 1.156	0.335
Pathological T stage (pT2, pT3)	0.589	0.178, 1.950	0.386	0.783	0.147, 4.153	0.773
Pathological Gleason’s score (**≦**6, 7, 8**≦**)	0.600	0.314, 1.144	0.121	0.495	0.179, 1.368	0.175
MRI measurements						
MUL	1.184	1.031, 1.359	0.017	1.227	1.011, 1.489	0.038*
BNW	1.617	1.181, 2.213	0.003	1.585	1.050, 2.393	0.028*
LMW	1.446	0.817, 2.561	0.205	1.087	0.486, 2.433	0.840

*Abbreviations*: *OR* Odds ratio, *CI* Confidence interval, *BMI* Body mass index, *BNW* Bladder neck width on the trigone side, *LMW* Levator muscle width, *MUL* Membranous urethral length

*On the multivariate analysis, MUL and BNW were associated with the period of early complete urinary continence

## Discussion

A previous study reported that the most significant factor affecting quality of life after RARP was urinary incontinence [3]. In the present study, urinary incontinence was identified in 15.1% patients at 1 year after RARP, which was consistent with previous reports [4–8]. Recent studies have reported that several anatomic characteristics are preoperative predictors of postoperative urinary incontinence, and surgical techniques are significantly associated with urinary incontinence after RARP [6, 9–12]. Recent advances in knowledge of the pelvic structure have led to increased understanding of the urinary continence mechanism [13]. In brief, the external striated sphincter is the main structure in maintaining a urethral closure pressure that is greater than bladder pressure. Several authors have evaluated the impact of pre- and postoperative MUL and found that continence recovery was slower in men with short MULs [10, 14, 18].

Postoperative MUL as measured on urethrovesicography has been reported to be the most important predictive factor for recovery of urinary continence in the early postoperative period after RARP, and it is greater with preservation of the neurovascular bundle, thus allowing early recovery of urinary continence [15]. However, the measurement of postoperative MUL and other anatomic characteristics of the pelvic floor shape on urethrovesicography seems to be inaccurate and limited; hence, we evaluated the postoperative MRI findings. The development of MRI has enabled more accurate measurement of anatomic characteristics of the pelvic floor shape. Furthermore, MRI allows a minimally invasive examination of anatomical structures at the points of radiation exposure and pain. MRI findings of MUL and LMW have been shown to be useful and independent predictors of postoperative continence recovery [9]. Pre- and postoperative MUL measured by MRI were significantly associated with urinary continence recovery after RARP, and pre- to postoperative MUL change was also associated with urinary continence recovery at 6 months after RARP [19]. Our results showed that postoperative MUL as evaluated on MRI was significantly associated with early urinary continence recovery, indicating that the residual MUL also influenced recovery of continence at 3 months after RARP.

Multiple intraoperative maneuvers have been proposed to improve urinary continence, including bladder neck preservation [20], and novel reconstruction of tissue around the vesicourethral anastomosis [21]. The posterior musculofascial plate plays a significant role as a dynamic support structure for the prostatomembranous urethra [22, 23]. Multilayer posterior reconstruction measured by postoperative MRI could potentially restore anatomic and functional defects more effectively and provide stronger posterior support to improve the recovery of urinary continence after RARP [24]. The posterior construction of bladder wall, including BNW thickness, also plays an important role as a dynamic support structure for early urinary continence recovery. Our results showed that postoperative BNW thickness on MRI was significantly associated with early urinary continence recovery. This study is the first to report that preserving MUL longer and making BNW thicker at surgery were important for early recovery continence after RARP.

Our study had several limitations. First, this study had a retrospective design and a relatively small sample size, and all surgeries were performed by the three different surgeons. However, the same RARP protocol was used during the study period. Second, urinary continence status was evaluated based on the number of pads that patients used, which is relatively subjective. Measurement of the 24-h pad weight is considered to be the most accurate test [25], but it is difficult to apply in practice. In this study, we evaluated whether patients used no pads or pads for protection; hence, the severity of urinary incontinence was measured by a self-reported number of pads used. Finally, other parameters affecting urinary incontinence might have been underestimated because we focused on the impacts of postoperative MUL, LMW, and BNW on urinary incontinence.

## Conclusions

Postoperative MUL as measured on MRI was significantly associated with early urinary continence recovery after RARP. Postoperative MUL was longer and BNW was thicker in patients who experienced early continence recovery (by 3 months after RARP). MUL and BNW on postoperative MRI were also related to the period of complete continence. Thus, surgeons’ efforts to preserve longer MUL and thicker BNW are recommended during surgery to achieve optimal continence outcomes after RARP.

## Data Availability

The datasets used and/or analyzed during the present study are available from the corresponding author on reasonable request.

## Abbreviations

BMI: Body mass index
BNW: Bladder neck width on the trigone side
CI: Confidence interval
LMW: Levator muscle width
MRI: Magnetic resonance imaging
MUL: Membranous urethral length
OR: Odds ratio
PSA: Preoperative prostate-specific antigen
RARP: Robotic-assisted radical prostatectomy
SD: Standard deviation
